# Unraveling the Role of *BRCA1* variants in Dysregulation of Transcriptional and Post-Transcriptional Mechanisms in Breast Cancer

**DOI:** 10.12669/pjms.40.6.8761

**Published:** 2024-07

**Authors:** Ayesha Isani Majeed, Sawdah Zinan, Rani Faryal

**Affiliations:** 1 Ayesha Isani Majeed, MBBS, MCPS, FRCR, MPH, MHPE, Ph.D. Department of Radiology and Breast Cancer Screening Programme, Pakistan Institute of Medical Sciences, Islamabad, Pakistan; 2 Sawdah Zinan, Department of Microbiology, Faculty of Biological Sciences, Quaid-i-Azam University, Islamabad, Pakistan; 3 Rani Faryal, Ph.D. Department of Microbiology, Faculty of Biological Sciences, Quaid-i-Azam University, Islamabad, Pakistan

**Keywords:** Breast cancer, miRNA, mRNA structure/stability, *BRCA1*, Variants

## Abstract

**Objective:**

To screen *BRCA1* gene variants and predict potential role of the identified variants in breast cancer.

**Method:**

This case-control study included two hundred and fifty breast cancer patients and equal healthy individuals from the Federal Breast Cancer Screening Centre, Pakistan Institute of Medical Sciences, Islamabad from March 2021- January 2023. Demographic data was collected through questionnaires and clinical data was assessed using mammograms, ultrasound, histopathology and immunohistochemistry reports. Polymerase chain reaction and Sanger sequencing approach were used to detect variants in *BRCA1* gene. *In-silico* analyses were carried out to predict mutation effect, miRNA binding site alterations and change in mRNA structure and stability.

**Results:**

Invasive ductal carcinoma was the most prevalent type of breast cancer. Old age [OR: 2.8149 (1.5995 to 4.9538) *p* value = 0.0003] and family history [OR: 4.3186 (1.7336 to 10.7581) *p* value = 0.001] were significant breast cancer risk. Six variants were identified. Two novel missense variants, Chr17:43082553A>T and Chr17:43093710A>T were predicted deleterious as these disrupted interaction with PALB2 and importin alpha’s NLS2 site, respectively. *In silico* analysis predicted the loss of hsa-miR-1179 binding site due to variant Chr17:43093220T>C. Moreover, four variants were predicted to affect the mRNA structure and stability.

**Conclusion:**

Two novel variants were predicted to be pathogenic. *In-silico* analysis predicted the loss of miRNA binding site along with change in mRNA secondary structure plus stability, possible mechanisms for oncogenesis. Further, expressional studies are required to confirm *BRCA1* gene dysregulation in breast cancer due to these variants.

## INTRODUCTION

Breast cancer is on the rise. According to GLOBOCAN (2020), 2.3 million new breast cancer cases and 685,000 deaths were reported. The future burden of breast cancer is predicted to be three million new cases and up to one million deaths by the year 2040.^1^ Pakistan holds the highest incidence of breast cancer, as one out of every nine women is at risk of breast cancer.^2^

Breast cancer is a multifactorial disease involving several exogenous and endogenous risk factors. *BRCA1*, a highly penetrant gene, plays a crucial role in breast cancer oncogenesis. Cells with the mutant *BRCA1* gene undergo uncontrolled multiplication and growth, resulting in tumour formation.^3^ Mutations and single nucleotide polymorphisms have a pivotal role in shaping the diversity of population and disease susceptibility. Previously, non-synonymous mutations were considered crucial however, emerging research suggests the role of synonymous mutations in the development of cancer due to impact on mRNA structure and stability, protein expression level, microRNA (miRNA) binding and splicing.^4^ miRNA regulates gene expression post-transcriptionally. Mutations in miRNA binding sites disrupt gene expression, leading to tumorigenesis.^5^ Furthermore, mutations can even impact the secondary structure of mRNA, affecting its stability and translational efficiency.^6^ Variants that occur within coding regions and miRNA binding sites can alter mRNA structure and stability which significantly affect the disease development.

In Pakistan, genetic testing of *BRCA1* is limited to private health clinics and few research studies have focused on the identification of *BRCA1* mutations.^7^-^8^ However, there remains a dire need to investigate the impact of variants on post-transcriptional regulations like miRNA binding sites as miRNAs are pivotal regulators of gene expression. Similarly, the variants can change mRNA stability and protein function.^4^ Thus, considering the burden of breast cancer in Pakistan and *BRCA1* being high penetrant gene, the current study was designed to screen participants for *BRCA1* gene variants. Additionally, *in-silico* approaches were used to decipher the impact of genetic changes on miRNA binding sites along with the structure and stability of transcripts in oncogenesis.

## METHODS

This retrospective study included 250 breast cancer patients and 250 healthy individuals. The patients were from Federal Breast Cancer Screening Centre, Pakistan Institute of Medical Sciences, Islamabad. Blood samples were collected in EDTA tubes after the consent and counselling of patients following the guidelines of Declaration of Helsinki by the World Medical Association from March 2021- January 2023. Blood samples after collection were kept in an icebox and brought immediately to the Molecular Medicine Laboratory at the Department of Microbiology in Quaid-i-Azam University, Islamabad and were stored at 4°C till further processing.

### Ethical Approval

The study was conducted after clearance from Bio Ethic Committee of Quaid-i-Azam University, Islamabad (Ref.no.IRB-QAU-183, dated January 22, 2019).

### Demographic and Clinical Data Acquisition

Demographic data was collected through structured questionnaires and in-person interviews. Mammograms, ultrasound, histopathology, immunohistochemistry (IHC) and breast imaging-reporting and data system (BI-RADS) were used to determine the histological type of cancer, cancer grade, scoring of the Progesterone Receptor (PR), Human Epidermal Growth Receptor 2 (HER2), Estrogen Receptor (ER) and tumour proliferation marker Ki-67%.

### Inclusion and Exclusion Criteria

All patients suffering from breast cancer were included whereas, in the case of controls, only healthy individuals were included. Patients suffering from cancers other than breast carcinoma were excluded.

### DNA Extraction and BRCA1 Gene Amplification

DNA extraction was performed by the phenol-chloroform method and the extracted DNAs were stored at -20°C. *BRCA1* gene reference sequence (ENSG00000012048) was retrieved and Primer 3 web software (http://primer3.ut.ee) was used for primer designing. Polymerase chain reaction (PCR) was carried out using reaction mixture containing Dream Taq Green PCR Master Mix (Thermo Scientific) through a thermocycler (Kyratec SC300G-R2, Australia). The PCR conditions used were same as mentioned by Shaukat M et al.^9^ The concentration of template ranged between 50-100ng and total volume of the PCR reaction mixture for each sample was 10µL. PCR amplicons were electrophoresed at 90 volts for 40 minutes (Clever scientific, UK) and visualized through Ultra-Violet Transilluminator.

### Mutational Screening and Sequencing

The amplified PCR products were subjected for single-strand conformational polymorphism analysis to screen variants based on variation in electrophoretic mobility. For this procedure, the amplified products were denatured by providing thermal shock in presence of 99% formamide. The polyacrylamide gel electrophoresis was carried out on 6% gel for amplified products with size <500bp and 8% for >500bp. Then these PCR amplicons were electrophoresed for 180 minutes at 120 volts. Banding patterns were observed and samples were sent for Sanger sequencing (Eurofins, USA). The sequencing results were analyzed through BioEdit (version 7.2.5) and Mutation Taster. Variants were interpreted using *BRCA1* gene reference sequence: ENSG00000012048 and were further analyzed using ClinVar and Variant Effect Predictor (VEP).

### In-silico Analysis

To predict the impact of non-synonymous genetic variations, PredictSNP, https://loschmidt.chemi.muni.cz/predictsnp/ was used. The alignment to miRNA to both mutated and reference sequence was checked through miRBase http://www.mirbase.org. Furthermore, Visual Gene Developer software (version 1.9) was used to predict the variant impact on the structure and stability of *BRCA1* mRNA.

### Statistical Analysis

Microsoft Excel and SPSS 20.0 were used for statistical analysis. Odds ratios were calculated and *p* values < 0.05 were considered statistically significant values.

## RESULTS

### Demographic and Clinical data

Most of the patients and healthy individuals belonged to Punjab province. Participants were categorized into three age groups. Age group III of ≥60 years posed a significant association with breast cancer risk [OR: 2.8149 (1.5995 to 4.9538) *p-value* =0.0003]. Majority had positive breastfeeding history. Similarly, 94.4% of patients were parous and few were nulliparous. Pre-menopausal patients were 50.8% while, 49.2% patients were menopausal. Patients with a family history of breast cancer showed significant breast cancer risk [OR: 4.3186 (1.7336 to 10.7581) *p* value = 0.001] as mentioned in Table-I.

**Table-I:** Demographic features of the patients and healthy individuals.

Categories	Cases n=250(%)	Controls n=250(%)	Odds Ratio (OR)	CI 95% Limited Unlimited	*p* Value
***Age distribution (years)***					
Group I: 28-40	55(22)	90(35)	0.5146	0.3464-0.7646	0.0010*
Group II:41-60	148(59.2)	141(56.4)	1.1217	0.7864-1.5999	0.5262
Group III: ≥61	47(18.8)	19(7.6)	2.8149	1.5995-4.9538	0.0003*
***Residence***					
Islamabad	50(20)	76 (30.4)	0.5724	0.3796-0.8630	0.0077*
Punjab	152(60.8)	124 (49.6)	1.5760	1.1052-2.2475	0.0120*
KPK	29(11.6)	22(8.8)	1.3599	0.7582-2.4392	0.3023
Sindh	1(0.4)	2(0.8)	0.4980	0.0449-5.5276	0.5702
Azad Kashmir	15(6)	21(8.4)	0.6960	0.3501-1.3838	0.3014
Gilgit Baltistan	2(0.8)	4(1.6)	0.4960	0.0900-2.7327	0.4206
Quetta	1(0.4)	1(0.4)	1.0000	0.0622-16.0775	1.0000
***Marital status***					
Married	244 (97.6)	245(98)	0.8299	0.2500-2.7555	0.7608
Unmarried	6(2.4)	5(2)	1.2049	0.3629-4.0005	0.7608
***Lactation***					
Yes	210(84)	220(88)	0.7159	0.4300-1.1919	0.1988
No	40(16)	30(12)	1.3968	0.8390-2.3255	0.1988
***Parity***					
Parous	236(94.4)	223(89.2)	2.1368	1.0917-4.1824	0.0267*
Nulliparous	14(5.6)	27(10.8)	0.4680	0.2391-0.9160	0.0267*
***Menopause***					
Pre-menopause	127(50.8)	144(57.6)	0.7600	0.5342-1.0815	0.1273
Menopause	123(49.2)	106(42.4)	1.3157	0.9247-1.8721	0.1273
***Family history***					
Yes	24 (9.6)	6 (2.4)	4.3186	1.7336-10.7581	0.0017*
No	226 (90.4)	244 (97.6)	0.2316	0.0930-0.5768	0.0017*

CI confidence interval,

* Significant value.

Invasive ductal carcinoma was more prevalent .Based on IHC reports, ER, PR, HER2 and Ki 67% were assessed. The most prevalent molecular subtype was luminal B (35.2%) followed by luminal A (30.8%). Considering the Nottingham grading system, Grade-II was the most diagnosed grade among patients. Mammograms and ultrasound reports revealed that half of the breast lesions were of category BI-RADS V (50.4%) as mentioned in Table-II.

**Table-II:** Classification of patient’s breast cancer type based on mammograms, histopathology BI-RADS and IHC.

Clinical Features	n=250 (%)
***Histologic type of Breast Cancer***	
Ductal carcinoma in-Situ	7(2.8)
Invasive ductal carcinoma	230(92)
Invasive lobular carcinoma	2(0.8)
Other rare types	11(4.4)
***Molecular Subtype***	
Luminal A	77(30.8)
Luminal B	88(35.2)
HER2 enriched	27(10.8)
TNBC	58(23.2)
***Grade***	
Grade-I	20(8)
Grade-II	195(78)
Grade 3	35(14)
***BI-RADS***	
BI-RADS III	3(1.2)
BI-RADS IV	58(23.2)
BI-RADS V	126(50.4)
BI-RADS VI	63(25.2)

### Mutational Screening and Sequencing

After analysis of Sanger sequencing, six variants (Reference: ENSG00000012048) in exon 10 (now called exon 11) of *BRCA1* gene were detected. Most of these mutations were detected in patients diagnosed with invasive ductal carcinoma. Variant Chr17:43092418A>G and Chr17:43093220T>C, were found both in patients and healthy individuals (Table-III).

**Table-III:** Identified variants of BRCA1 gene and predicted changes in its protein.

S. No.	Physical location of SNP	Variant site	Coding Consequence by VEP	Amino acid change	Predicted effect of Variants	Participants Patients/controls		SNP status in current study

						(P)	(C)	
1	Chr17:43093710 A>T	NLS2	Missense	Lys607Asn	Deleterious	2	0	Novel
2	Chr17:43092507G>A	Interaction with RAD51	Missense	Met1008Ile	Neutral	6	0	rs1800704
3	Chr17:43092418A>G	Interaction with RAD51	Missense	Glu 1038Gly	Neutral	15	10	rs16941
4	Chr17:43082553A>T	Interaction with PALB2	Missense	Asn1403Ile	Deleterious	5	0	Novel
5	Chr17:43093220T>C	Interaction with RAD50	Synonymous	Leu771 Leu	-	21	9	rs16940
6	Chr17: 43094127G>A	c-Myc binding site	Synonymous	Lys468Lys	-	5	0	rs1555591782

### In-silico Analysis

Variant Chr17:43082553A>T and Chr17:43093710A>T were predicted with pathogenic effects disrupting the function of BRCA1 (Table-III). The variants were further analyzed if any alterations in miRNA binding, in variant Chr17:43093220T**>**C deletion of binding site for hsa-miR-1179 was seen. The *in-silico* analysis predicted variant Chr17:43092507G>A, Chr17: 43094127G>A, Chr17:43092418A>G and Chr17:43093220T>C compromised the mRNA structure and stability.Table-IV

**Table-IV:** In-silico analysis of impact of identified mutations of* BRCA1* on mRNA structure, mRNA stability and miRNA binding sites in transcripts

Variant	Gibbs energy Kcal/mol	mRNA structure change	Stability Effect	miRNA ID	miRNA Alignment (R)	miRNA Alignment (M)
Chr17:43093220T>C	-48.40(R)	Changed	Decreased	hsa-miR-1179	Aligned	Not aligned
	-50.40(M)					
Chr17:43093710A>T	-45.10(R)	No	No effect	hsa-miR-4703-5p	Aligned	Aligned
	-45.10(M)					
Chr17:43092507G>A	-34.70(R)	Changed	Increased	hsa-miR-6734-5p	Aligned	Aligned
	-32.60(M)					
Chr17:43092418A>G	-36.00(R)	Changed	Decreased	hsa-miR-3167	Aligned	Aligned
	-43.40(M)					
Chr17:43082553A>T	-39.00(R)	No	No effect	hsa-miR-4751	Aligned	Aligned
	-39.00(M)					
Chr17: 43094127G>A	-40.80(R)	Changed	Increased	hsa-miR-4680-5p	Aligned	Aligned
	-38.10(M)					

(R) Reference sequence; (M) Mutated sequence.

## DISCUSSION

This retrospective case-control study presents the screening of *BRCA1* variants and predicted impact of these identified variants on the function of BRCA1 along with if any dysregulation of transcriptional or post-transcriptional mechanisms in breast cancer. Demographic risk factors like age and family history were found to increase risk of breast cancer in this study. Genetic testing of *BRCA1* revealed six variants. Among these variants, two variants were predicted with deleterious effect, four variants compromised the mRNA structure and stability while one variant was predicted to alter the miRNA binding site.

Pakistan has the highest age-standardized breast cancer incidence rates among Asian countries.^10^ Most of the patients in this study were of age group II (41-60 years) however, age greater than 60 years in this study was found to be significant risk for breast cancer development [OR: 2.8149 (1.5995 to 4.9538) *p* value = 0.0003]. In most of the Asian countries,^10^-^12^ the average age for incidence of breast cancer range between 40-59 years while in the Western world, the average age is 60-70 years.^13^ The age of a woman at which she gives birth to her first child and the number of children is also risk factors for breast cancer. Null parity increases the risk of breast cancer,^14^ however, in this study 94.4% of patients were parous. Similarly, a study conducted by Ahmed F et al.^15^ in Pakistan reported 93% breast cancer patients as parous. About 84% patients were breastfeeding their children, contrary to earlier report that breastfeeding for one year reduces breast cancer by 4.3%.^16^ Menopause leads to the termination of the reproductive period among females and generally occurs in between 45-55 years of age, however, late menopause in women is a great risk of breast cancer due to longer exposure of hormones. Pre-menopausal and menopausal patients found in this study were 50.8% and 49.2% respectively. Similarly, Ahmed F et al.^15^ reported 54.74% cancer in pre-menopausal patients. Patients with a family history of breast cancer were found to have high risk of breast cancer (OR: 4.3186 [1.7336 to 10.7581] *p* value =0.0017). Similarly, Majeed AI et al.^7^ reported 5% of the patients as familial. In familial cases, inherited genetic changes increase the risk of breast cancer.

Invasive ductal carcinoma was found as the most prevalent type. Likewise, Khan ME et al.^17^ reported 95.6% of the patients with invasive ductal carcinoma. According to the American Cancer Society (2023), invasive ductal carcinoma represents approximately 70-80% of all breast cancer cases. Luminal B was found in 35.2% patients in the current study. A study conducted in Pakistan by Alam S et al.^18^ also reported Luminal B with greater frequency than Luminal A. Luminal B sub-type is the most prevalent type among breast cancer patients in the current study, it has worst prognosis and high metastatic potential as compared to Luminal A. The majority of patients were diagnosed with Grade-II (78%). These results are consistent with Ahmed F et al.^16^ study, where 73.2% of breast cancer patients had Grade-II. High grade tumours have worst prognosis and are associated with increased mortality than Grade-I.

Overall, six genetic variants were detected in *BRCA1*. In coding region, change in nucleotide influence the secondary structure of mRNA, which disrupts the translation process and protein expression.^19^ The predicted mRNA stability of variant Chr17:43092507G>A and Chr17: 43094127G>A was increased while for variants Chr17:43092418A>G and Chr17:43093220T>C stability of *BRCA1* mRNA was reduced as their Gibbs free energy changed.

All the identified variants were subjected to *in-silico* analysis for binding of regulatory miRNAs. According to miRBase, miRNA hsa-miR-1179 did not bind to the target *BRCA1* transcript of variant Chr17:43093220T>C. Upregulation of miR-1179 in breast cancer serves as a tumour suppressor and is also involved in suppression of Notch signaling pathway in breast cancer.^20^ The non-binding of hsa-miR-1179 with *BRCA1* mRNA signifies loss of an anti-oncogenic regulator and chance of oncogenesis.

The functional changes were predicted in these variants. A novel variant Chr17:43082553A>T(Asn1403Ile) was identified in coiled-coil domain of BRCA1, which is the interaction site of partner and localizer of BRCA2 (PALB2), and was predicted as deleterious. PALB2 is a molecular adaptor between the both BRCA1 and two proteins. Due to this mutation, change is BRCA1-PALB2 interaction can lead to defective homologous recombination (HR). Such impaired HR repair can lead to genomic instability and tumourigenesis.^21^ Another novel variant Chr17:43093710 A>T (Lys607Asn) was detected in nuclear localization sequence (NLS2) and was predicted to have deleterious effect. NSL1 and NSL2 are recognized by importin-α and mediate the transport of BRCA1 from cytosol to nucleus. A mutation in such sequences can disrupt the interaction of importin-α with BRCA1, due to which BRCA1 cannot translocate into nucleus and will remain in cytosol. Thus, the DNA repair ability of BRCA1 in the nucleus will be compromised, which can lead to accumulation of unrepaired mutations and abnormalities within the chromosome.^22^ The overall finding of the study shows that due to mutations in *BRCA1*, dysregulation of both transcription and post-transcription occurs. Such variants of *BRCA1* can change DNA repair function of BRCA1 leading to oncogenesis.

This study identifies two novel variants in *BRCA1* gene. Additionally, this study not only highlights the impact of variants on protein function but also depicts the disruption of miRNA binding, mRNA structure and stability. Furthermore, these findings will help to develop diagnostic biomarkers and further treatment regime to reduce the breast cancer burden in Pakistan. However, large-scale population-based studies that recruit patients from different regions across the country are required to overcome the selection biasness. *In-silico* tools provide valuable insights to predict the impact of genetic variants but such variants need to be validate in wet lab.

## CONCLUSION

Novel variants identified in this study are predicted to have altered expression of BRCA1. However, expressional based research is required to further elucidate and confirm the functional impact of the identified *BRCA1* genetic variation for its role in breast oncogenesis and for its potential therapeutic development. Additionally, there is need to investigate other tumour suppressor genes including *BRCA2* in breast cancer cases. Also, multi-omics approach should be applied to gain a more holistic molecular understanding of breast cancer.

### Authors Contribution:

**AIM:** Conceived, designed, did statistical analysis of the manuscript. **SZ:** Did data collection and experimental work. **RF:** Designed the research work, editing, review, approved and responsible for integrity of research.
